# Supplementary material for: Laminar shear stress inhibits inflammation by activating autophagy in human aortic endothelial cells through HMGB1 nuclear translocation
Source: Commun Biol. 2022 May 6;5:425. doi: 10.1038/s42003-022-03392-y (PMC9076621; doi:10.1038/s42003-022-03392-y)
Supplement: Supplementary file 2 — Supplementary Information [file 42003_2022_3392_MOESM2_ESM.pdf]

Laminar shear stress inhibits inflammation by activating autophagy in human aortic endothelial cells through HMGB1 nuclear translocation

Supplementary Information

Qingyu Meng [1], Luya Pu [1], Mingran Qi[1], Shuai Li [1], Banghao Sun[1], Yaru Wang[1], Bin Liu [2]* and Fan Li [1,3,4,5,6]*

[1]Department of Pathogenobiology, The Key Laboratory of Zoonosis, Chinese Ministry of Education, College of Basic Medicine, Jilin University, Changchun, China

[2]Cardiovascular Disease Center, The First Hospital of Jilin University, Changchun, China

[3]Engineering Research Center for Medical Biomaterials of Jilin Province, Jilin University, Changchun, China

[4]Key Laboratory for Health Biomedical Materials of Jilin Province, Jilin University, Changchun, China

[5]State Key Laboratory of Pathogenesis, Prevention and Treatment of High Incidence Diseases in Central Asia, Xinjiang, China

[6]The Key Laboratory for Bionics Engineering, Ministry of Education, Jilin University, Changchun, China

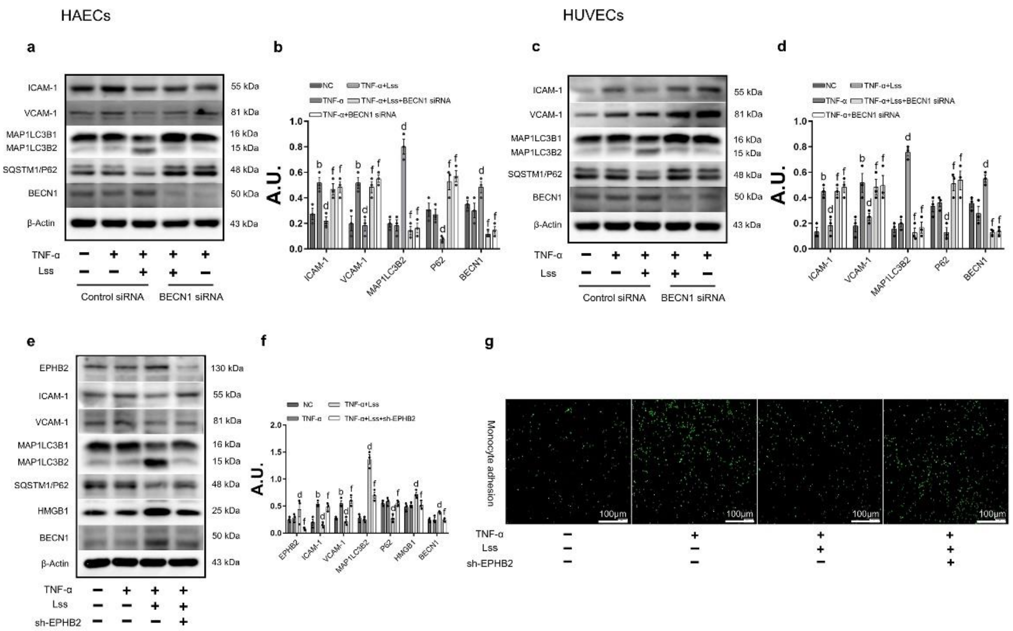

Fig. S1. **LSS inhibits the inflammatory response in endothelial cells by activating autophagy.** a-b. Western Blot shows the effect of BECN1 siRNA on anti-inflammatory effect of LSS in HAECs. c-d. Western Blot shows the effect of BECN1 siRNA on anti-inflammatory effect of LSS in HUVECs. e-g. Western Blot and confocal microscopy shows the effect of sh-EPHB2 on anti-inflammatory effect of LSS. Data are presented as mean ± SEM of three independent experiments. $^{a}P < 0.05$, $^{b}P < 0.01$ vs. NC group; $^{c}P < 0.05$, $^{d}P < 0.01$ vs. TNF-α group. $^{e}P < 0.05$, $^{f}P < 0.01$ vs. TNF-α+LSS group. AU, arbitrary units.

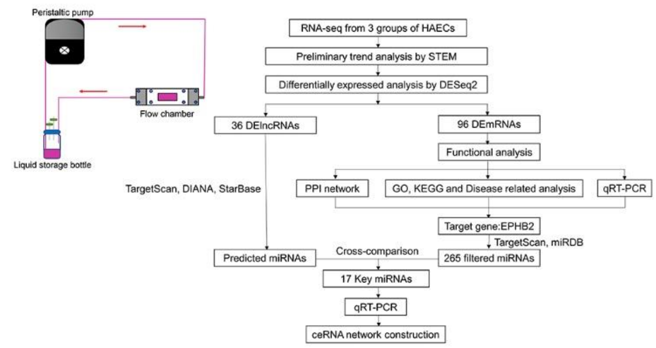

Fig. S2. **Flowchart of the research showing the steps involved in construction of the LSS-related LOC107986345/miR-128-3p/EPHB2 regulatory axis.**

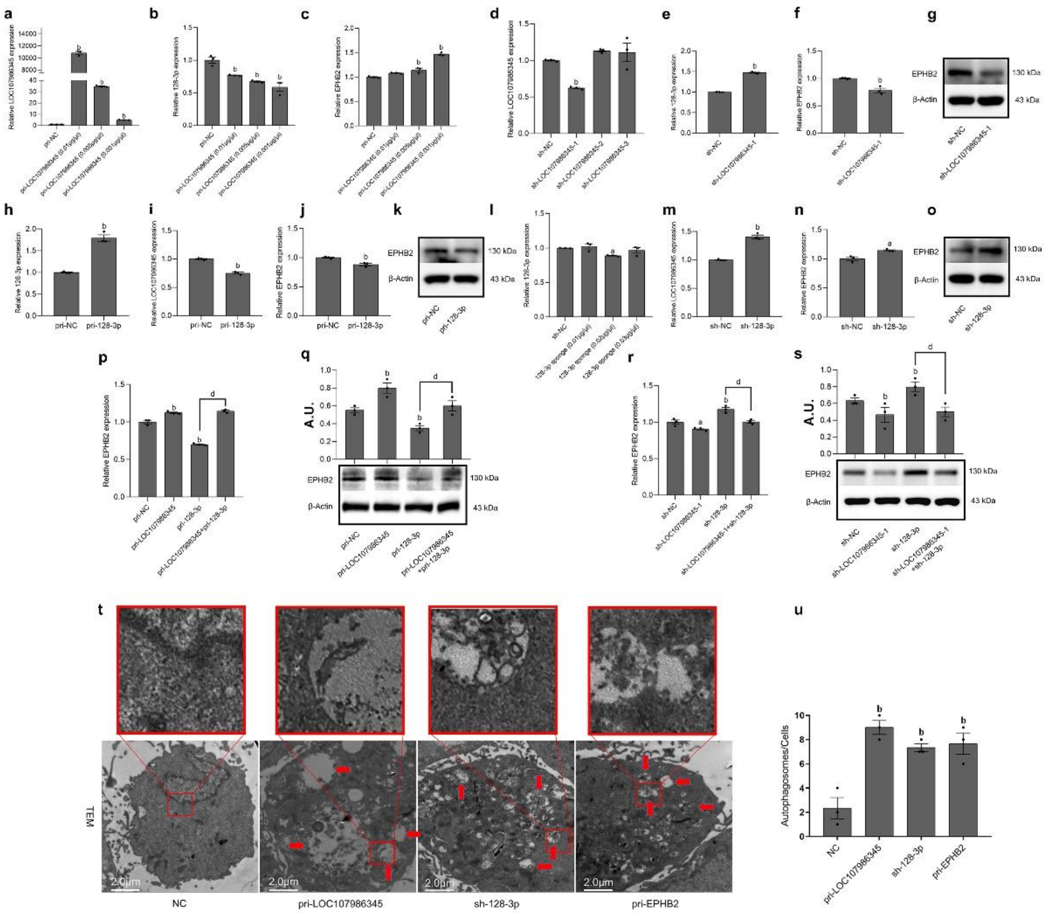

Fig. S3. **Construction of the LSS-related LOC107986345/miR-128-3p/EPHB2 network.**

a-c. qRT-PCR shows the expression of LOC107986345, miR-128-3p and EPHB2 after pri-LOC107986345. d-g. qRT-PCR and Western Blot shows the expression of LOC107986345, miR-128-3p and EPHB2 after sh-LOC107986345. h-k. qRT-PCR and Western Blot shows the expression of LOC107986345, miR-128-3p and EPHB2 after pri-miR-128-3p. l-o. qRT-PCR and Western Blot shows the expression of LOC107986345, miR-128-3p and EPHB2 after sh-miR-128-3p. p-q. qRT-PCR and Western Blot shows the expression of EPHB2 in endothelial cells co-transfected with LOC107986345 and miR-128-3p overexpression plasmids. r-s. qRT-PCR and Western Blot shows the expression of EPHB2 in endothelial cells co-transfected with LOC107986345 and miR-128-3p knockdown plasmids. t-u. TEM shows autophagosomes and autophagolysosomes in HAECs. Arrows indicate autophagosomes. Data are presented as mean ± SEM of three independent experiments. $^{a}P < 0.05$, $^{b}P < 0.01$ vs. NC group; $^{c}P < 0.05$, $^{d}P < 0.01$ vs. pri-miR-128-3p or sh-miR-128-3p group. AU, arbitrary units.

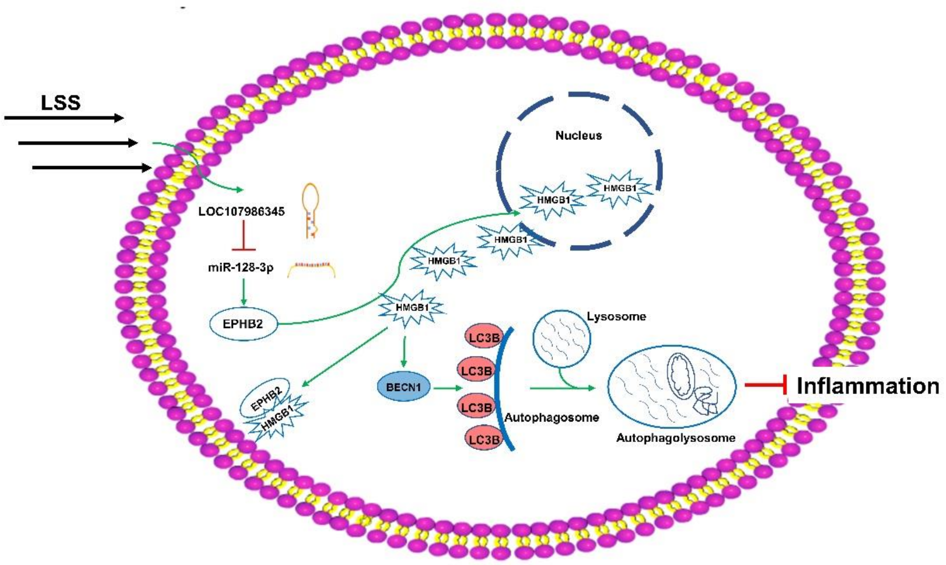

Fig. S4. **Schematic diagram of anti-inflammatory effect of LSS through LOC107986345/miR-128-3p/EPHB2 axis in an autophagy-dependent pathway which induced by nuclear translocation of HMGB1 in endothelial cells.**

When endothelial cells were treated with LSS, LSS-sensitive lncRNA, LOC10798635, combines with miR-128-3p to increase the expression of EPHB2. Up-regulation of EPHB2 expression can promote nuclear translocation of HMGB1, which can activate the autophagy pathway of endothelial cells. Autophagy activation significantly inhibited the increased expression of ICAM-1, VCAM-1, COX-2, and MMP-9 caused by TNF-α stimulation. The arrows represent promotion, whereas the inverted T means inhibition.

Table S1**. 17 key miRNAs after the intersection of miRNAs that potentially bind to EPHB2 (Table 3) and miRNAs predicted by 36 DE lncRNAs (Table 4).**

miRNA	lncRNA
hsa-miR-4306	LOC105374005, AC008505.1
hsa-miR-216a-3p	LOC107986196, LOC107986345
hsa-miR-128-3p	LOC107986196, LOC107986345
hsa-miR-27a-3p	LOC107986196, LOC107986345
hsa-miR-30c-5p	LOC107986345, MALAT1
hsa-miR-3681-3p	LOC107986345
hsa-miR-495-3p	LOC101930665, SNHG20
hsa-miR-9-5p	LOC101930275
hsa-miR-185-5p	AC008505.1
hsa-miR-4644	AC008505.1
hsa-miR-23c	AC008505.1, MALAT1
hsa-miR-23a-3p	AC008505.1, MALAT1
hsa-miR-150-5p	MALAT1
hsa-miR-204-5p	MALAT1
hsa-miR-211-5p	MALAT1
hsa-miR-23b-3p	MALAT1
hsa-miR-5688	SNHG20

Table S2. **Sequence information for LOC107986345**

LncRNA	FASTA
LOC107986345,<br>XR_001742403.1	**>XR_001742403.1 PREDICTED: Homo sapiens uncharacterized LOC107986345 (LOC107986345), ncRNA**<br>AAATTTTGCTTTCAGCAATTTTCCATTTTTTTTCAGCATACTTATATCACAATGAGGCAACCAAATATGAA<br>TCCTGAGAATGAAGATGGAAAATGAATATTTTAATCCAATATCGAGTTGTTCTTTCAACATTTTTAGTTT<br>TAGTTTTAACTGAAAATATAAAGTTACATAATTGTGTGAGGAATAATTTTTGCAGAGAAATTTTTAAAAA<br>TTCACAAAAGGAAAAAGAAACCTTTACCATGAGTCTATAACATTGAAAGACTGTGTTTAAGTGATTTTAA<br>TAAAGCCAAACCAACACTTTCAGCAGGAACTAAATGGCAGCATCTGATTTCACGCTGATACATATGTATT<br>AGTGTGGTGCTCACTTTTACCCTTCATTTTACCTTGGGTCTTGCCATTTTCTTTCTATTTTTACGTTTTT<br>TTTTTTTTTTTTCTTTTGGAAGAGGTAATTTTTAGGGAGAAAAAAACACTTTTTCCTCATAGGTCGATTT<br>AAAATGTTGGCCTTACCTTAATCTCCTCTCTCAAACTCAATCCACTATGTAATGGGTCAACATACTTTTC<br>TGTGAAGGACCAGATAGTAAATATTTTAGTCTTTGCAGGCCATATGGTCTCTGTTGCCAGCTCTTCCATT<br>GTGGTGTGAAAGCAGCCATAGACAACACAGAAATGAATAAGTGTAACTGTTCCAATAAAAACAGATGATA<br>TGTTGAATTTAGCTCACAGAGTTTAGCTTGCTGCCCCTGCAGGAGGCCTTTGGAGTAAAAGCTTCCTGAG<br>AGGAGGAATTTTTGTCTTTTTTGCTCAAGTTCTAGCTCCAGTACCTAAAATAGTGCCTGTCACGTAGGTA<br>TTGATGAATATTTGAACCTGTTGAACATACACCTAAAATAAAACATTTGGCAAGATACAGTACTACACAA<br>TTTGGAAAACACTTGGCTCCCATAGAAATCAAAGCCTTCCTGAGTAATTAATTATTTGGCCTGATGATGA<br>ATTACTGTGCCTGAGATGATAGAGCTAATTTATTTTTCAATTCACTCAGGGGACACACGTTATTTTCACT<br>GTGAATTTGGTTAAAATGAAAAGATTTCCTGCTCTAAGTCCTGGATAGACCTTTATGTAATAGCATACTC<br>TTCACTCTTTTTGAATCGCATGCAGTTGTCACACTGGATGATTTCCAGACAGAGGTTCCAAGTCTTTCAT<br>CATGTTTGGGTTAAAGGCCTCATTAACATACTAGTCCTGCCATTTGAGTCTGTTCTCTTCACGGAATATT<br>TTCACCTGAATCAGTGGGTATAATTCATCAGTGTCTGGTTGCTTCAAGTTATTTTTCTTAATGCTGATGT<br>TAATGCATGCCCATCTTTATGCCTCAACACATTGCACAATGAAAAACAAAAATTATTTTGGAAGACACAG<br>CTGACATCTATATGAAATATTCAGTATCAGTGATTGAACTTCAGCAAGCTCCTGTGGCCAGCAGGGTTTT<br>ACGGAGGTGCAACTGCTCTCCCATGATCACTTATCATAAAGCCAGAGACAATTGGGCCAACGTAGCATCT<br>TGCCTGCTTTTGTGTACAAAATATAAATAGAATAGAGCAAACAAGGAAAAATAGTCCCGCAGCTGGAAGG<br>CAACTTTAAAGAAAAATGATGTTCATAGCTATCTTGCCAGTAGCTGGCAAAGCAGTTTTTATAAGACATA<br>TTTAATGTTTACAACCACTTGGAGGCTGGGGAAGGAGTAATACTTTGCAAATATGTTTAATGTTGTGAAC<br>CACTCTGTGGCCGAGAAGCAACTCCGGCTAAAATATATTTAATGCTTAGAGTCATTTGGTGGTTTCGAAA<br>AAAAAGAAACTTCTTGTTAATTTTTAACATTTATTGCTGATCAGTGGCTGCTAAAGTAACTATCAGTACA<br>GCATGGTTTATGTTTAGGGACACGCTGTGCTGCTACTAACACAATTCATACTCTTCTCTCAAGGCTACAC<br>AAAAAGTGTCAATAGCATTACCTTTGCAATTGCACCACTAATAAAAACACAGGAATCAAACAGACGAATA<br>AAATGCTCCCTGCCCACACAGCCATAACATCCTATGGCCCTGAAAATAGTCCTGCCAAACTGCGTGCAGT<br>GGACACTTCACGGCTGGCACAGGAGAGATCACAAGGCCTTGCTCAATTTCATCAAAAGTGTTAAAGCGAC<br>TCAGCAGATTGTGAAGCACAAGTGGAAGCTGATAATTGGTGTTTCTTACAAATCAACGGCTTGTCTCCAC<br>ACATCAAGGTGAGAAAATCAATGTAAAAGAGGAGAAATAACCCACACAACTACATACAACTAGGAAATGA<br>TAGTTGGATCTGAACAAAAGGATATGCACTCTTAACAAGTTCAACATCCAAGTACTATTGCAAGTACAAG<br>CTGTATTTCATGGAAAAA

Table S3. **shRNA/ siRNA target sequences**

Name	Target Sequnece (5'-3')
sh-LOC107986345-1	GGGTTAAAGGCCTCATTAACA
sh-LOC107986345-2	GCTCTCCCATGATCACTTATC
sh-LOC107986345-3	GCAACTGCTCTCCCATGATCA
Negative control (sh-LOC107986345)	CCTAAGGTTAAGTCGCCCTCG
miR-128-3p sponge (sh-128-3p)	aaagagacccaacactgtga
sh-EPHB2-1	ACGAGAACATGAACACTAT
sh-EPHB2-2	TGAACAGTATCCAGGTGAT
sh-EPHB2-3	ACCCGACTACACCAGCTTTAA
Negative control (sh-EPHB2)	GTTCTCCGAACGTGTCACGTT
HMGB1 siRNA	GGACAAGGCCCGTTATGAA
BECN1 siRNA	GGAGACATTATGGAGAGATT
Control siRNA	CCTAAGGTTAAGTCGCCCTCG

Table S4. **Primer sequences**

Gene Name	Forward Primer	Reverse Primer
EPHB2	GTACCTGGCAGACATGAACTAT	GTATCGTCCTCTAGAAAGCGTG
LOC107986345	CCTGCCCACACAGCCATAACATC	GTGCCAGCCGTGAAGTGTCC
LOC107986196	AGGATGTCCTTGAGAGGCTAACCC	GGTGCTCCTCATGCTGTCGTTC
has-miR-128-3p	CGCCGTCACAGTGAACCGGTCTCTTT	
hsa-miR-4306	CGCGCGTGGAGAGAAAGGCAGTA	
hsa-miR-216a-3p	CGCGCTCACAGTGGTCTCTGGGATTAT	
hsa-miR-27a-3p	CGCGTTCACAGTGGCTAAGTTCCGC	
hsa-miR-3681-3p	CGCGCACACAGTGCTTCATCCACTACT	
hsa-miR-495-3p	CGCGCGAAACAAACATGGTGCACTTCT	
hsa-miR-9-5p	GGCGCGCGTCTTTGGTTATCTAGCTGT	
hsa-miR-185-5p	CGCGTGGAGAGAAAGGCAGTTCCTGA	
hsa-miR-4644	CGCGCGTGGAGAGAGAAAAGAGACAGA	
hsa-miR-23c	CGCGCATCACATTGCCAGTGATTACCC	
hsa-miR-23a-3p	CGCCGATCACATTGCCAGGGATTTCC	
hsa-miR-150-5p	CGCTCTCCCAACCCTTGTACCAGTG	
hsa-miR-30c-5p	CGCCGCGCTGTAAACATCCTACACTCT	
hsa-miR-204-5p	CGCCGTTCCCTTTGTCATCCTATGCCT	
hsa-miR-211-5p	CGCTTCCCTTTGTCATCCTTCGCCT	
hsa-miR-23b-3p	CGCGATCACATTGCCAGGGATTACCAC	
hsa-miR-5688	CGCGCGCGCTAACAAACACCTGTAAA	
β-actin	GGCCAACCGCGAGAAGATGAC	GGATAGCACAGCCTGGATAGCAAC
U6	CTCGCTTCGGCAGCACA	AACGCTTCACGAATTTGCGT

Fig. S5. **All uncropped western blots in this study.**

Fig.1

a-LC3

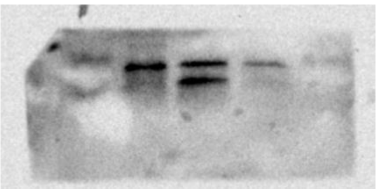

a-P62

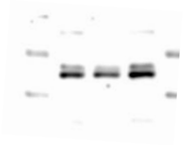

a-β actin

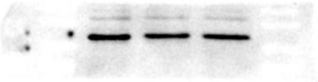

c-LC3

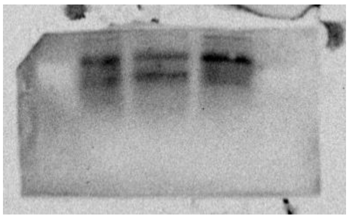

c-P62

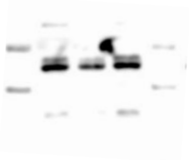

c-β actin

e-LC3

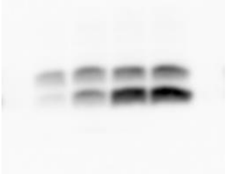

e-β actin

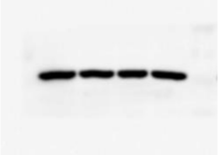

g-LC3

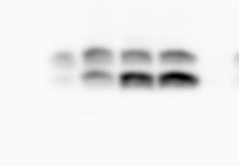

g-β actin

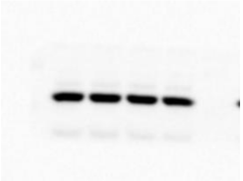

i-COX2

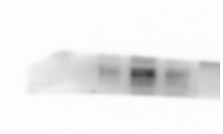

i-ICAM-1

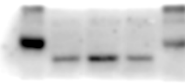

i-LC3

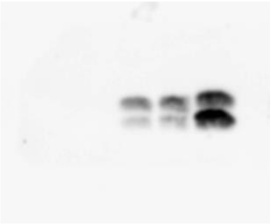

i-MMP9

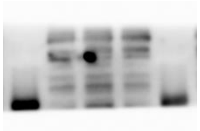

i-P62

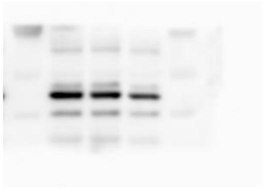

i-VCAM-1

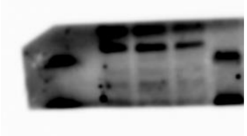

i-β actin

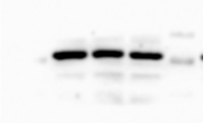

k-COX2

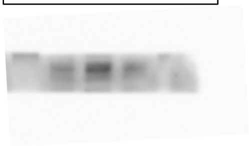

k-ICAM-1

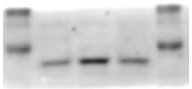

k-LC3

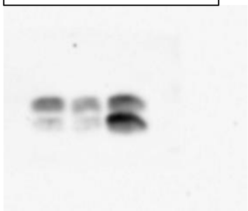

k-MMP9

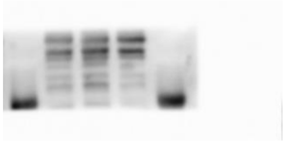

k-P62

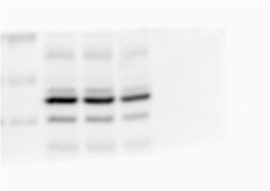

k-VCAM-1

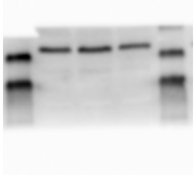

k-β actin

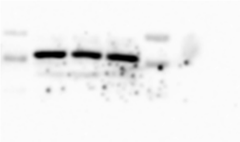

m-COX2

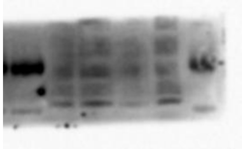

m-ICAM

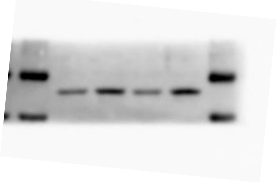

m-LC3

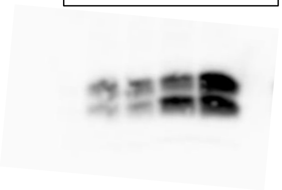

m-MMP9

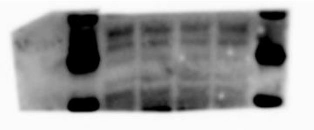

m-P62

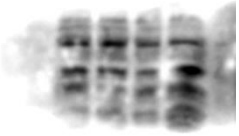

m-VCAM-1

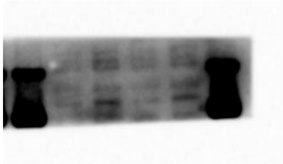

m-β actin

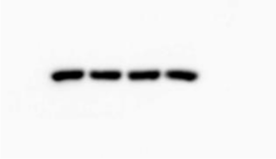

o-COX2

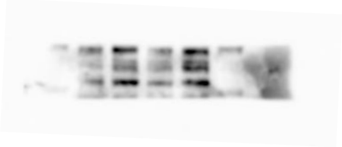

o-ICAM

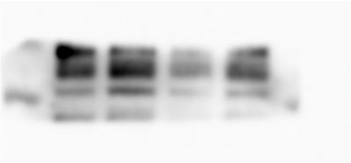

o-LC3

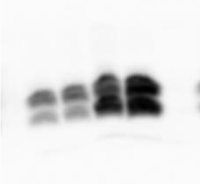

o-MMP9

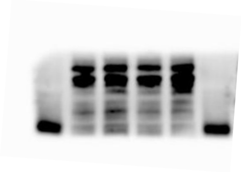

o-P62

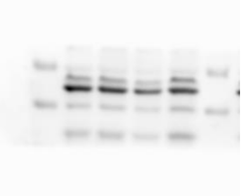

o-VCAM-1

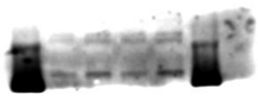

o-β actin

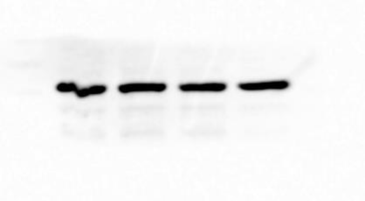

Fig.2

k-EPHB2

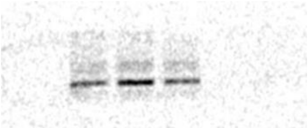

k-GAPDH

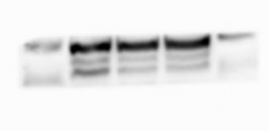

Fig.3

b-EPHB2

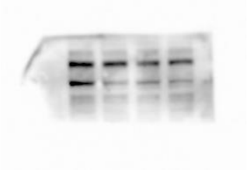

c-EPHB2

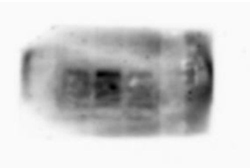

c-LC3

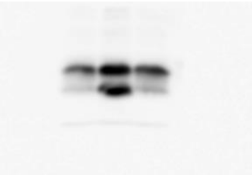

c-P62

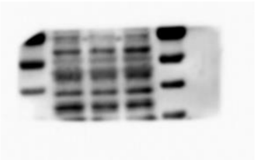

c-β-actin

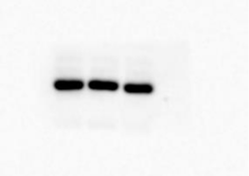

i-BECN

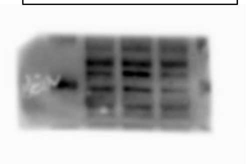

i-Cyto-HMGB1

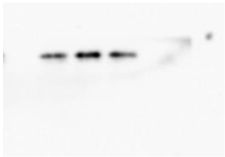

i-Lamin-B

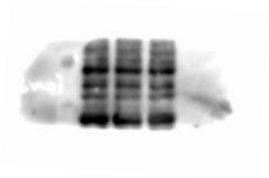

i-Nuc-HMGB1

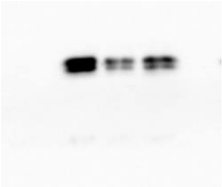

i-β-actin

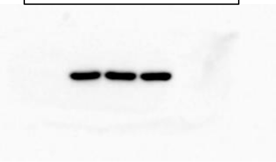

l-co-IP-EPHB2

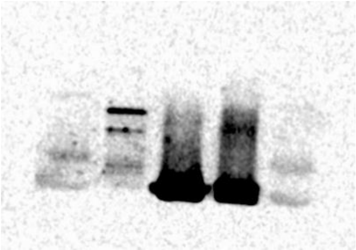

l-co-IP-HMGB1

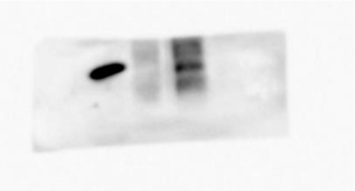

Fig.4

a-BECN-1

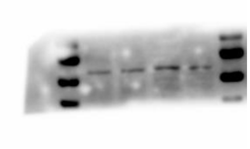

a-HMGB1

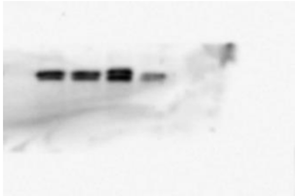

a-ICAM-1

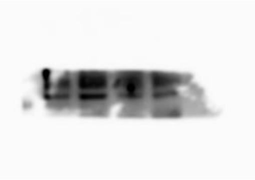

a-LC3

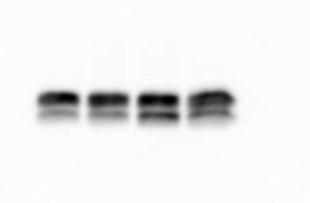

a-P62

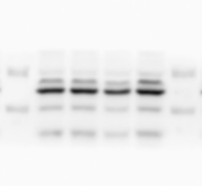

a-VCAM-1

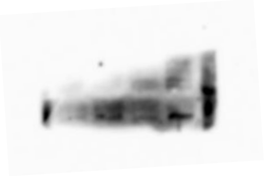

a-β actin

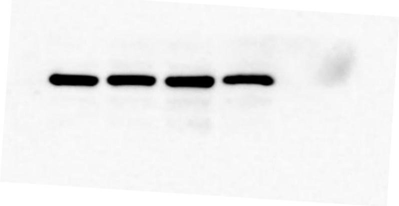

e-BECN-1

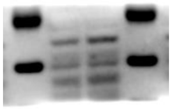

e-Cyto-HMGB1

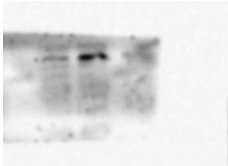

e-EPHB2

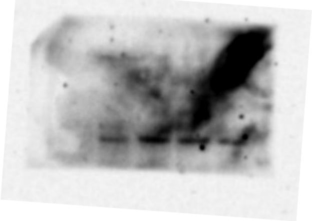

e-Lamin-B

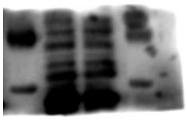

e-Nuc-HMGB1

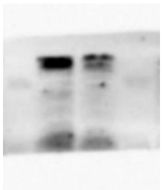

e-β actin

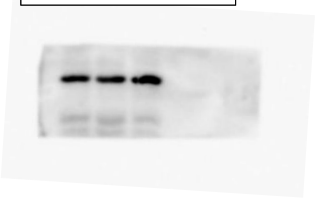

f-co-IP-EPHB2

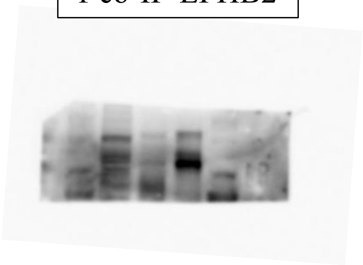

f-co-IP-HMGB1

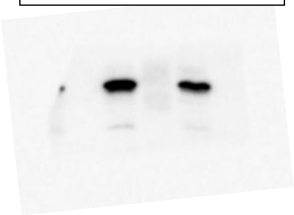

h-BECN-1

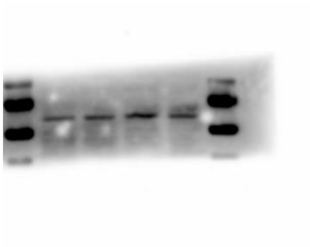

h-EPHB2

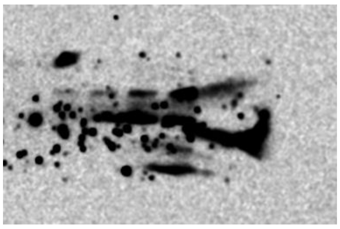

h-HMGB1

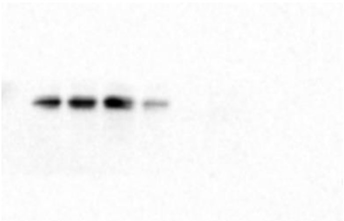

h-ICAM-1

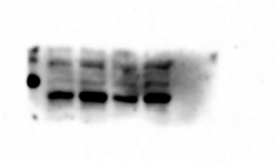

h-LC3

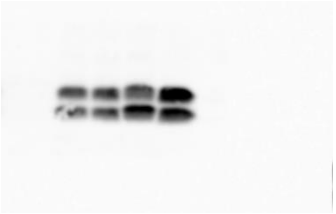

h-P62

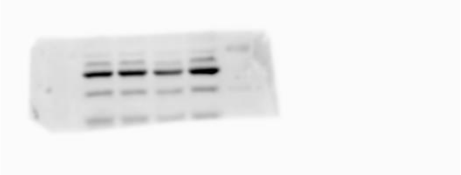

h-VCAM-1

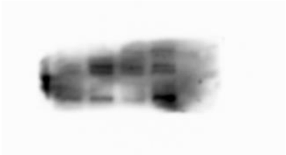

h-β actin

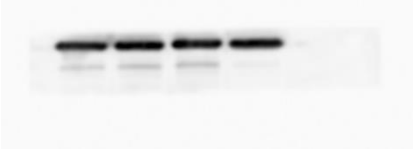

k-BECN1

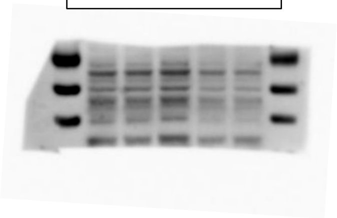

k-HMGB1

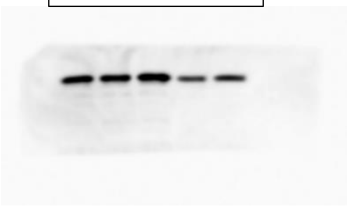

k-ICAM-1

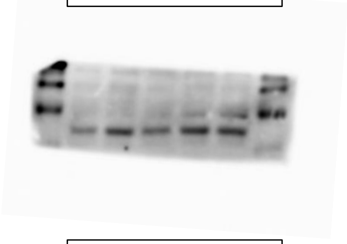

k-LC3

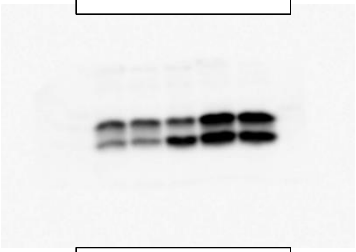

k-P62

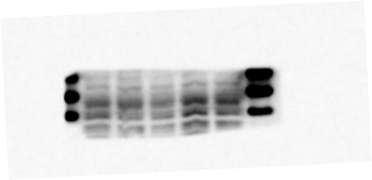

k-VCAM-1

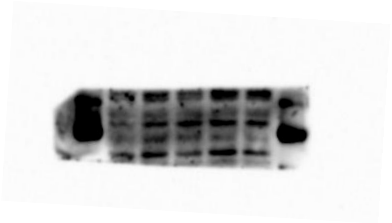

k-β actin

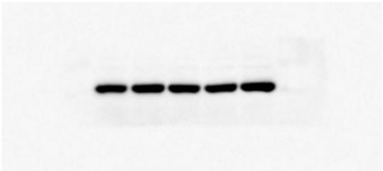

m-BECN1

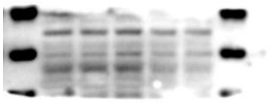

m-HMGB1

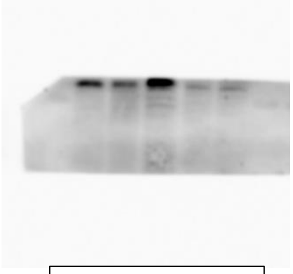

m-ICAM-1

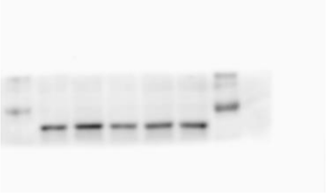

m-LC3

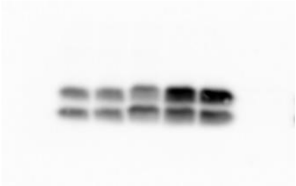

m-P62

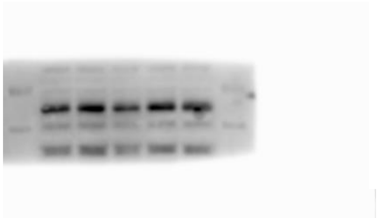

m-VCAM-1

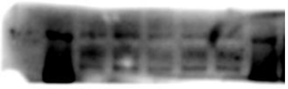

m-β actin

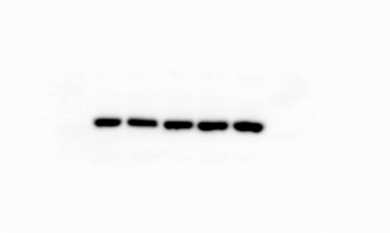

Fig.5

n-IP-AGO2

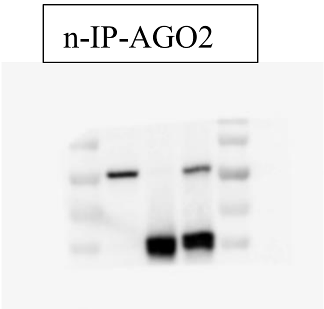

Fig.6

c-LC3

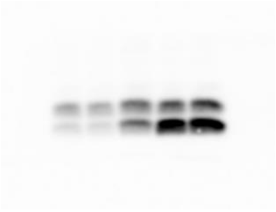

c-β actin

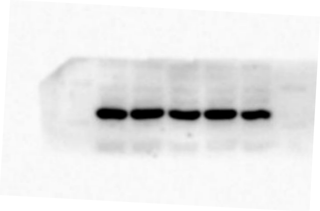

d-LC3

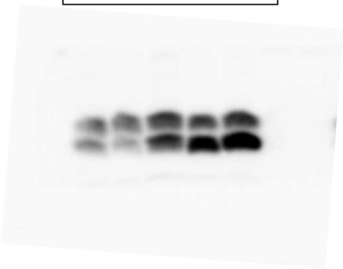

d-β actin

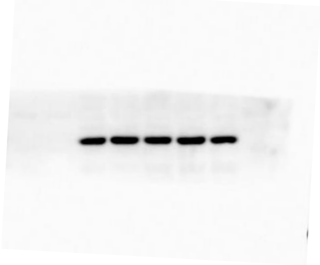

e-LC3

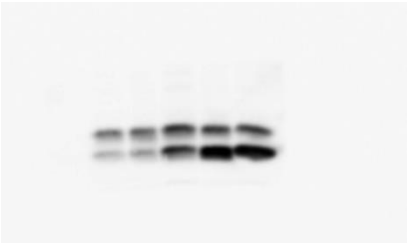

e-β actin

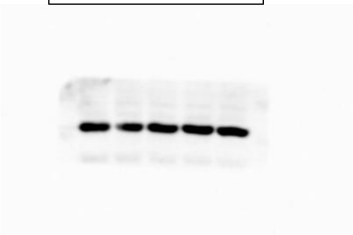

h-EPHB2

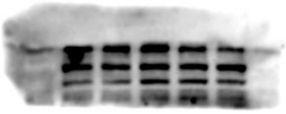

h-ICAM-1

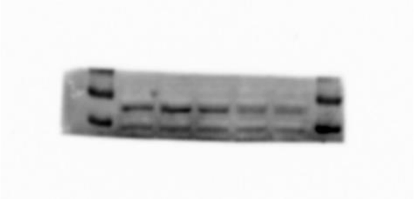

h-LC3

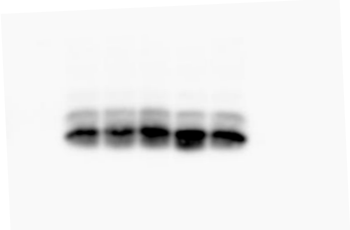

h-P62

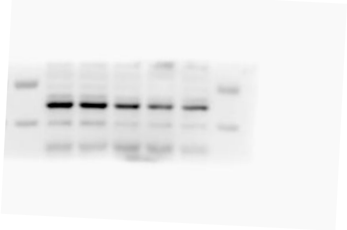

h-VCAM-1

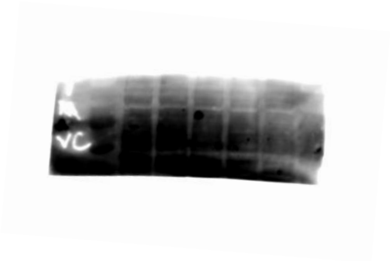

h-β actin

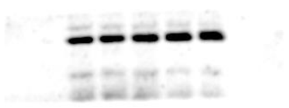

j-BECN1

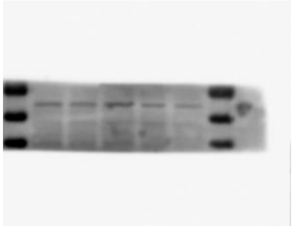

j-HMGB1

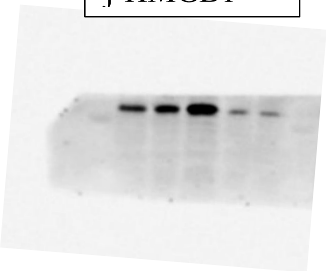

j-ICAM-1

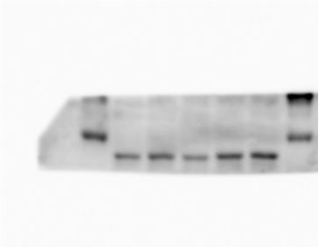

j-LC3

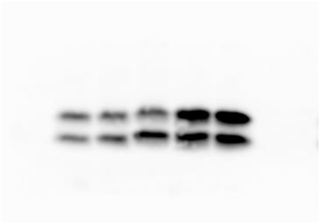

j-P62

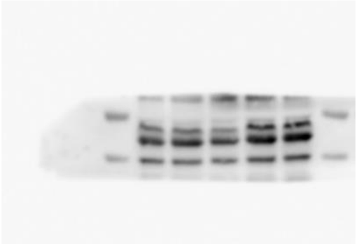

j-VCAM-1

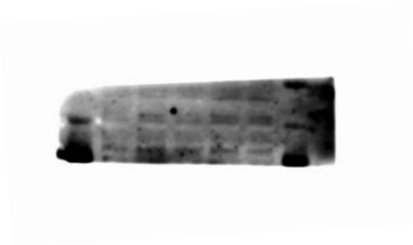

j-β actin

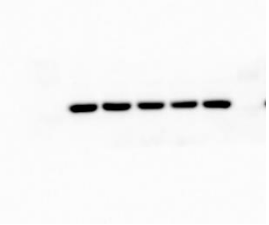

l-BECN1

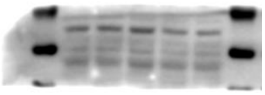

l-HMGB1

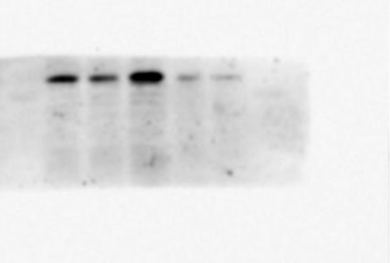

l-ICAM-1

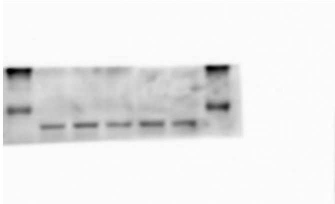

l-LC3

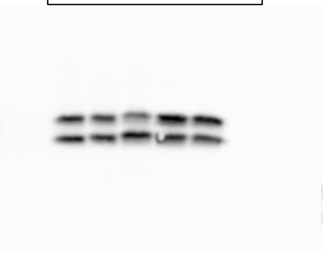

l-P62

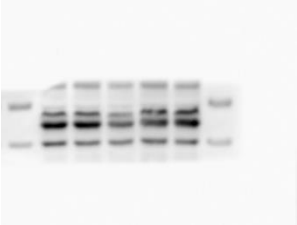

l-VCAM-1

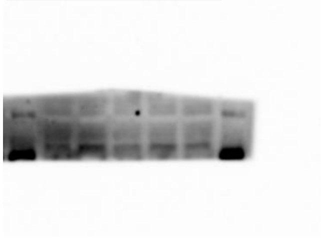

l-β actin

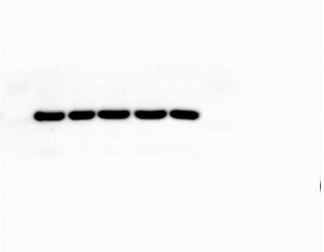

l-P62

Fig.S1

a-BECN1

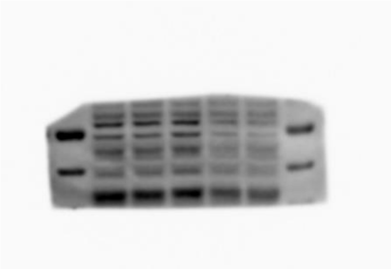

a-ICAM-1

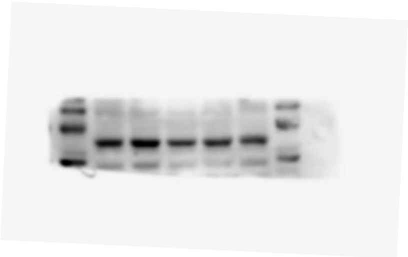

a-LC3

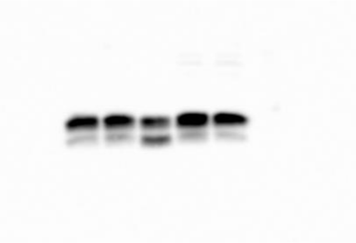

a-P62

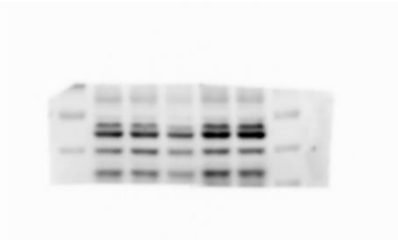

a-VCAM-1

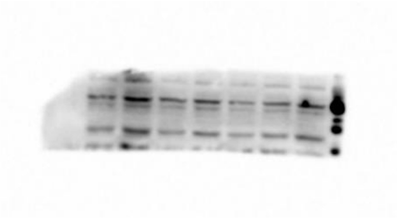

a-β actin

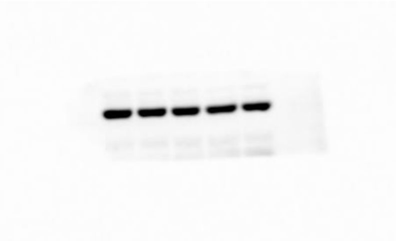

c-BECN1

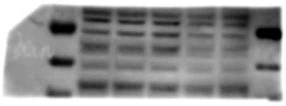

c-ICAM-1

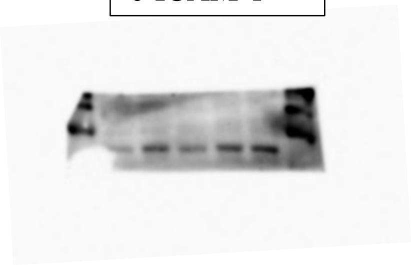

c-LC3

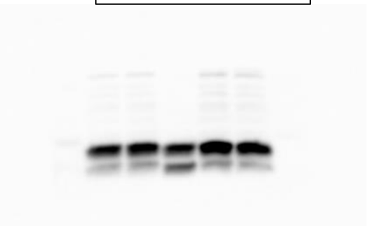

c-P62

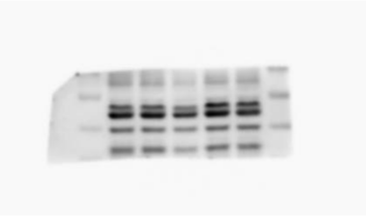

c-VCAM-1

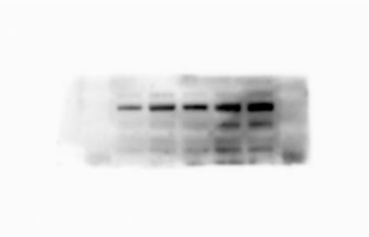

c-β actin

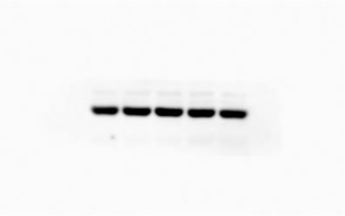

e-BECN1

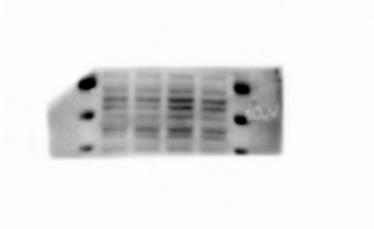

e-EPHB2

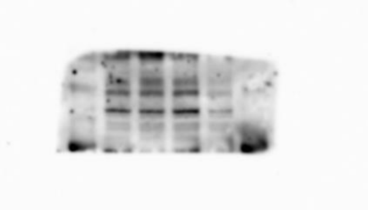

e-HMGB1

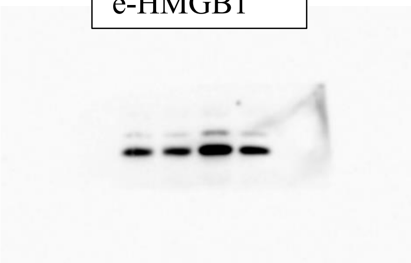

e-ICAM-1

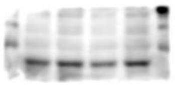

e-LC3

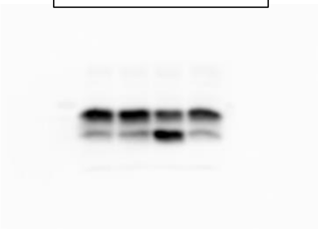

e-P62

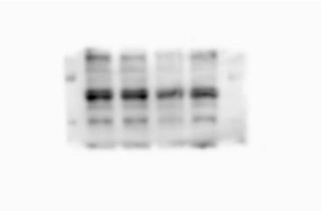

e-VCAM-1

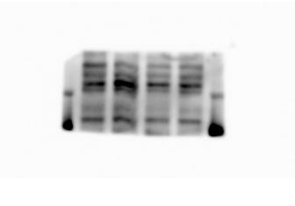

e-β actin

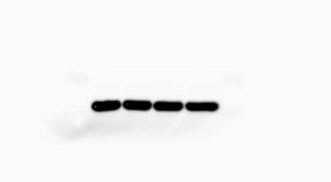

e-P62

Fig.S3

g-EPHB2

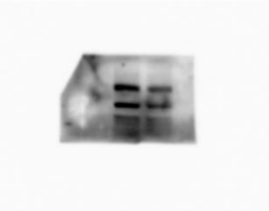

g-β-actin

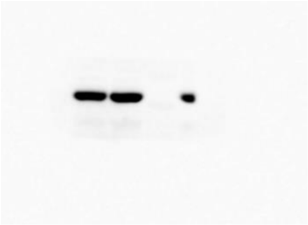

k-EPHB2

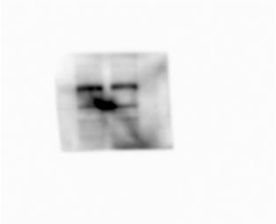

k-β-actin

o-EPHB2

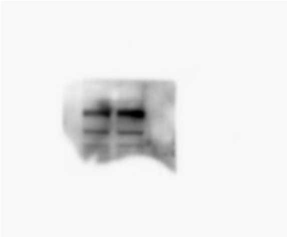

o-β-actin

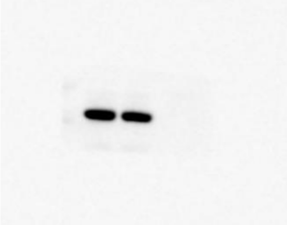

q-EPHB2

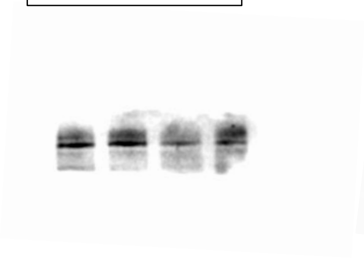

q-β actin

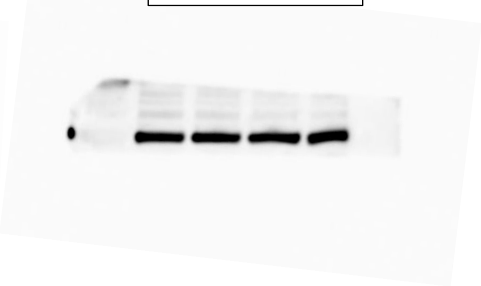

s-EPHB2

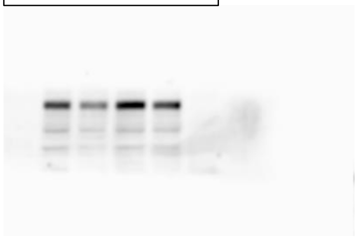

s-β actin
