# Supplementary material for: Laminar shear stress inhibits inflammation by activating autophagy in human aortic endothelial cells through HMGB1 nuclear translocation
Source: Commun Biol. 2022 May 6;5:425. doi: 10.1038/s42003-022-03392-y (PMC9076621; doi:10.1038/s42003-022-03392-y)
Supplement: Supplementary file 3 — Description of Additional Supplementary Files [file 42003_2022_3392_MOESM3_ESM.pdf]

Description of Additional Supplementary Files

File name: Supplementary Data 1

Description: All source data underlying the graphs and charts in figures have been uploaded as Supplementary Data in the Excel format.

File name: Supplementary Data 2

Description: Table 1 in Excel format.

File name: Supplementary Data 3

Description: Table 2 in Excel format.

File name: Supplementary Data 4

Description: Table 3 in Excel format.

File name: Supplementary Data 5

Description: Table 4 in Excel format.
