# Supplementary material for: Laminar shear stress inhibits inflammation by activating autophagy in human aortic endothelial cells through HMGB1 nuclear translocation
Source: Commun Biol. 2022 May 6;5:425. doi: 10.1038/s42003-022-03392-y (PMC9076621; doi:10.1038/s42003-022-03392-y)
Supplement: Supplementary file 5 — Reporting Summary [file 42003_2022_3392_MOESM5_ESM.pdf]

nature portfolio

Corresponding author(s): Bin Liu and Fan Li

Last updated by author(s): Apr 13, 2022

Reporting Summary

Nature Portfolio wishes to improve the reproducibility of the work that we publish. This form provides structure for consistency and transparency in reporting. For further information on Nature Portfolio policies, see our Editorial Policies and the Editorial Policy Checklist.

Statistics

For all statistical analyses, confirm that the following items are present in the figure legend, table legend, main text, or Methods section.

n/a	Confirmed	
☐	☒	The exact sample size (*n*) for each experimental group/condition, given as a discrete number and unit of measurement
☐	☒	A statement on whether measurements were taken from distinct samples or whether the same sample was measured repeatedly
☐	☒	The statistical test(s) used AND whether they are one- or two-sided<br>*Only common tests should be described solely by name; describe more complex techniques in the Methods section.*
☒	☐	A description of all covariates tested
☒	☐	A description of any assumptions or corrections, such as tests of normality and adjustment for multiple comparisons
☐	☒	A full description of the statistical parameters including central tendency (e.g. means) or other basic estimates (e.g. regression coefficient) AND variation (e.g. standard deviation) or associated estimates of uncertainty (e.g. confidence intervals)
☐	☒	For null hypothesis testing, the test statistic (e.g. *F*, *t*, *r*) with confidence intervals, effect sizes, degrees of freedom and *P* value noted<br>*Give P values as exact values whenever suitable.*
☒	☐	For Bayesian analysis, information on the choice of priors and Markov chain Monte Carlo settings
☒	☐	For hierarchical and complex designs, identification of the appropriate level for tests and full reporting of outcomes
☒	☐	Estimates of effect sizes (e.g. Cohen's *d*, Pearson's *r*), indicating how they were calculated

Our web collection on statistics for biologists contains articles on many of the points above.

Software and code

Policy information about availability of computer code

Data collection	HAECs used for whole-transcriptome sequencing were divided into three groups: the static-state group (Static group); the 12-dyn/cm2 LSS group that continued to act on endothelial cells for 12 h (LSS group); and the 12-dyn/cm2 LSS group, which, after 12 hours of treatment, were returned to steady-state culture for 24 h (After LSS group).
Data analysis	All data in this study are presented as mean±standard error of the mean. Statistical analyses were performed using IBM SPSS Statistics 20.0 software. The data in different groups were analyzed by one-way analysis of variance (ANOVA) followed by Tukey's post-hoc test. A P value of <0.05 was considered statistically significant.

For manuscripts utilizing custom algorithms or software that are central to the research but not yet described in published literature, software must be made available to editors and reviewers. We strongly encourage code deposition in a community repository (e.g. GitHub). See the Nature Portfolio guidelines for submitting code & software for further information.

Data

Policy information about availability of data

All manuscripts must include a data availability statement. This statement should provide the following information, where applicable:

- Accession codes, unique identifiers, or web links for publicly available datasets
- A description of any restrictions on data availability
- For clinical datasets or third party data, please ensure that the statement adheres to our policy

The data in this study was obtained by our research group through high-throughput sequencing, and has been uploaded to the GEO database (GSE199709).

Field-specific reporting

Please select the one below that is the best fit for your research. If you are not sure, read the appropriate sections before making your selection.

☒ Life sciences ☐ Behavioural & social sciences ☐ Ecological, evolutionary & environmental sciences

For a reference copy of the document with all sections, see nature.com/documents/nr-reporting-summary-flat.pdf

Life sciences study design

All studies must disclose on these points even when the disclosure is negative.

Sample size	Data are presented as mean ± SEM of three independent experiments.
Data exclusions	No data in this study were excluded from the analyses.
Replication	All experiments were repeated more than three times, and each repetition was in line with expectations.
Randomization	All samples were collected randomly.
Blinding	All data is collected blindly

Reporting for specific materials, systems and methods

We require information from authors about some types of materials, experimental systems and methods used in many studies. Here, indicate whether each material, system or method listed is relevant to your study. If you are not sure if a list item applies to your research, read the appropriate section before selecting a response.

Materials & experimental systems

n/a	Involved in the study	
☐	☒	Antibodies
☐	☒	Eukaryotic cell lines
☒	☐	Palaeontology and archaeology
☒	☐	Animals and other organisms
☒	☐	Human research participants
☒	☐	Clinical data
☒	☐	Dual use research of concern

Methods

n/a	Involved in the study	
☒	☐	ChIP-seq
☒	☐	Flow cytometry
☒	☐	MRI-based neuroimaging

Antibodies

Antibodies used	EPHB2 (ab216629, Abcam, Great Britain), ICAM-1 (ab282575, Abcam, Great Britain), VCAM-1 (ab134047, Abcam, Great Britain), COX-2 (ab179800, Abcam, Great Britain), MMP-9 (ab76003, Abcam, Great Britain), MAP1LC3B2 (ab192890, Abcam, Great Britain), SQSTM1/p62 (ab109012, Abcam, Great Britain), HMGB1 (ab79823, Abcam, Great Britain), BECN1 (ab210498, Abcam, Great Britain), Lamin B (ab16048, Abcam, Great Britain), β-actin (ab8227, Abcam, Great Britain), and GAPDH (ab128915, Abcam, Great Britain); goat anti-rabbit immunoglobulin G (IgG) (ab6721, Abcam, Great Britain)
Validation	https://www.abcam.cn/

Eukaryotic cell lines

Policy information about cell lines

Cell line source(s)	Department of Pathogenobiology, Basic Medicine College of Jilin University (Changchun, China)
Authentication	None of the cell lines used were authenticated.
Mycoplasma contamination	All cell lines tested negative for mcoplasma contamination.
Commonly misidentified lines (See ICLAC register)	The cell line used in this study is HAECs, which is an important component of the aortic intima and is closely related to vascular diseases.
